# A rapid and low-cost method for genomic DNA extraction from the cyanobacterium *Synechocystis*

**DOI:** 10.1093/biomethods/bpaa011

**Published:** 2020-06-13

**Authors:** Dale J A Harrison, Elinor P Thompson

**Affiliations:** School of Science, University of Greenwich, Chatham Maritime, Kent ME4 4TB, UK

**Keywords:** DNA extraction, *Synechocystis*, cyanobacterium

## Abstract

A two-step method is reported for preparation of genomic DNA from the model cyanobacterium *Synechocystis* that can be performed with minimal equipment and reagents in about an hour. High yields of genetic material can be obtained (200–450 ng/μl) with reasonable purity. A further ethanol precipitation step can be included but is not necessary if template is simply required for polymerase chain reaction (PCR) or digestion. This new protocol is helpful for amplification of genes of interest in early-stage research projects and for low throughput screening of transformants. It is more reliable than colony PCR of *Synechocystis* cultures, and less involved and cheaper than existing clean-DNA preparation methods. It represents an unusually simple and reliable extraction protocol for the growing body of research making use of this cyanobacterium.

## Introduction

The increasing interest in cyanobacteria for biotechnology follows their long history as models for the chloroplast [1]. *Synechocystis* sp. PCC 6803 became a pre-eminent model organism in photosynthesis research as it was the first photoautotrophic organism to have its complete genome sequence published [2] and, in addition, it is naturally transformable [3]. This, and early crystal structures of photosystems from the closely related cyanobacterium *Thermosynechococcus elongatus*, were key to better understanding of photosynthesis and its regulation [4, 5]. Following the early arrival of *Synechocystis* in molecular biology and photosynthetic research, it maintains its position as an experimentally amenable photoautotroph in the laboratory by virtue of its increasing use in systems biology and biotechnology. An unusually large research base of genomic, biochemical, and physiological data mean that cyanobacteria are considered to provide an excellent genetic framework for synthetic biology [6, 7] and for drug development [8–10] by virtue of their native anticancer and proapoptotic compounds, along with their overproduction of phenylpropanoid precursors [11, 12]. Their use in sustainable bioenergy research has been an area of particular activity [13–15] including production of bioethanol [16] or hydrogen [17], and they have been explored as workhorses for bioplastic production (for review, see Katayama *et al*. [18]).

Because hundreds of studies using this model organism have been published annually for decades, it is also possible to evaluate and compare data from different laboratories and strains for informed planning and scale-up. Meanwhile, methods for use with *Synechocystis* have been optimized for many years. DNA extraction remains a practical challenge for many people engaged in cyanobacterial research, however. Sufficient yield and quality are required for repeated use of genomic DNA as template in PCR, in order to feed amplicons through mutations, insertions, or deletions in cloning vectors. A rapid and efficient mechanism is also required for the analysis of DNA from transformants. It is noticeable in performing rapid DNA extraction from transformed *Arabidopsis thaliana* compared with transformed *Synechocystis* that the former has more reliable “quick and dirty” methods [19, 20]. Rapid and reliable extraction of genetic material, ideally with low time and financial commitment and limited chemical hazards, would be of benefit to many *Synechocystis* projects. Existing cyanobacterial DNA extraction procedures, however, tend to use harmful solvents, labile enzyme stocks, and time-consuming protocols.

The need to break the resistant *Synechocystis* cell adds an extra step to kit-based methods. The multilayered cell wall and S layer [21] is disrupted in existing procedures by enzymatic lysis [e.g., lysozyme [22], multicomponent buffers [23], or physical means (e.g., glass beads [24])]. The procedure outlined below therefore minimizes the number of steps for the process and avoids costly reagents and multicomponent buffers, by reducing glass bead breaking steps, then adapting one of the simplest methods used for DNA extraction from plants (the “Shorty” prep [20]). PCR and restriction digests on the extracts tested showed it would be possible to use this straightforward protocol to increase efficiency within many *Synechocystis* research projects.

## Materials and methods

### Cyanobacterial culture

*Synechocystis* sp. PCC 6803 (GT strain; gift from Prof. CW Mullineaux, Queen Mary University of London) was cultured using BG11 [25] supplemented with 10 mM sodium bicarbonate, and, for plates, with 10 mM 2-[(2-hydroxy-1,1-bis[hydroxymethyl]ethyl) amino]ethanesulfonic acid, 3 g/l sodium thiosulfate, and 15 g/l agar, with incubation conditions of 30°C, 148 rpm, 24 h light (intensity, 10 umol photons/m^2^/s).

### Rapid DNA extraction

The 40 ml of overnight and long-term *Synechocystis* cultures (of ∼2 × 10^8^ cells/ml) was pelleted in 50 ml sterile centrifuge tubes (Fisher, Hampton, USA) at 4000 *g* for 5 min. The supernatant was removed from each tube and the pellet was resuspended in sterile deionized water, and centrifugation repeated to remove residual medium. The tube containing the washed cell pellet was placed on ice and resuspended in 5 ml extraction buffer [200 mM Tris-HCl pH8.0 (Sigma, Darmstadt, Germany); 0.4 M lithium chloride (Fisher); 25 mM EDTA pH 8 (Sigma); 1% w/v SDS (Applichem, Ottoweg, Germany); pH 9.0]. Approximately 200 μl of sterile acid-washed glass beads (150–212 µm; Sigma) was added to the resuspended pellet, the tube was vortexed for 30 s and then returned to ice for 30 s. This step was repeated 5 times. After centrifuging at 3000 *g* for 15 min at 4°C, the supernatant was gently taken up into a sterile 10 ml syringe (Beckton Dickinson, Franklin Lakes, USA) and filtered through a sterile 0.2 μm filter (Minisart; Sartorius, Göttingen, Germany). From 5 ml pellet in buffer, ∼4 ml of filtrate was collected in a 5 ml tube. This was split into five aliquots of 800 μl in 1.5 ml microcentrifuge tubes for alcohol precipitation of DNA, when required. This was achieved by adding 600 μl of ice-cold isopropanol (Fisher) and immediate mixing by pipetting. Samples were then centrifuged at 16 000 *g* for 20 min at 4°C, and the supernatant removed carefully so as not to disturb the pellet. Tubes were left to air dry for 15 min then 200 μl of TE buffer (2 mM Tris-HCl pH 8.0; 1 mM EDTA pH 8.0) was added for resuspension of the pellet. Resuspended material was transferred from each tube to the next in turn, to resuspend each pellet sequentially, and all DNA were collected in one 200 μl aliquot.

### Optional purification step

The 10 μl of sterile 3 M sodium acetate (pH 5.2; Sigma) was added to 100 μl of genomic DNA extract and vortexed to mix. The 300 μl of ice-cold absolute ethanol (Fisher) was added before vortexing again and incubating the tube at −20°C for 2 h. Samples were centrifuged at 16 000 *g* for 30 min at 4°C, and the supernatant was removed. The pellet was washed by adding 200 μl of room temperature 70% ethanol, centrifuging at 10 000 *g* for min at 4°C, and removing the supernatant. The pellet was again left to air dry for 10 min, before resuspending it in 50 μl of TE pH 8.0. Samples, once resuspended, were centrifuged for 3 min at 5000 *g* and the supernatant was carefully transferred to a fresh, sterile 1.5 ml tube.

### DNA analysis

Purity of DNA was assessed using A_260_/A_230_ and A_260_/A_280_ values (NanoDrop 2000C spectrophotometer; ThermoFisher, Waltham, MA, USA; Supplementary Fig. S1). DNA quality and quantity were also checked by gel electrophoresis and compared with a bacteriophage lambda digest.

### PCR and enzyme digestion

Whether DNA quality was appropriate for use as a template in PCR was assessed in reactions for a standard housekeeping gene (130 bp of the 16 s rRNA gene; 5′-AGCGTCCGTAGGTGGTTATG-3′ and 5′-CTACGCATTTCACCGCTACA-3′), and two further test open reading frames with cloning primers containing mismatches for enzyme cut sites (1024 bp product from 5′-GCCggattcAGGCCCGTGAATTTCTTAAA-3′ and 5′-CAAggtaccGATATAGTCCGATAATTTGCT-3′; 620 bp product from 5′-CTAgaattcATTTTTGCTGTAGTAATGC-3′ and 5′ AAAGTCAcggccgGCCCCTTCT-3′). PCR was carried out using 1 μl of the extracted DNA (with or without purification), DreamTaq polymerase (ThermoFisher) and RNase/DNase free water (HyPure; GE Life Sciences, Marlborough, MA, USA) in a 25 μl total volume in 0.2 ml PCR tubes (Starlabs). Cycles were designed according to standard practice, with initial 5 min denaturation at 95°C, annealing for 1 min at temperatures set according to primer Tm, and a final extension period of 7 min at 72°C.

Restriction digests were set up according to standard practice, using *Nhe*I and appropriate buffer (New England Biolabs, Ipswich, MA, USA).

## Results

**Figure 1:**
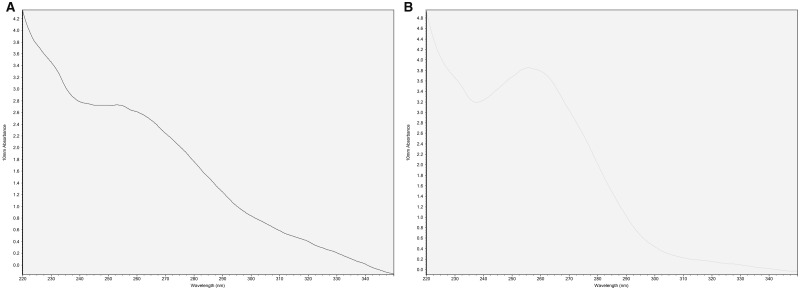
Analysis of DNA isolated from *Synechocystis.* (**A**) δ extract, (**B**) δ purified.

**Figure 2:**
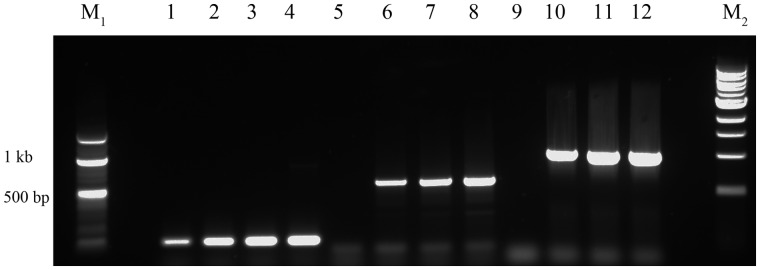
Replicate PCR using α, β, γ, and δ from crude extract as template, respectively. Lanes 1–4, PCR for 130 bp 16S rRNA product; 5–8, PCR for 620 bp product; PCR for 9–12, 1024 bp product. M_1_, 100 bp ladder, M_2_, 1 kb ladder.

DNA was successfully isolated using the rapid extraction method from new and stock cultures. Three out of four low purity extracts (by A_260_/A_230_ and A_260_/A_280_ ratios; Table 1, Fig. 1) were of sufficient quality for PCR amplification of products of various sizes including using primers with mismatches (Fig. 2). PCR was also satisfactory from extractions from nonexponentially growing stock cultures (Supplementary Fig. S2).

**Table 1:** DNA concentration and quality following rapid extraction

Sample name	A_260_/A_230_	A_260_/A_280_	Yield (ng/μl)
α	0.62	1.43	197.6
β	0.53	1.48	212.6
γ	0.41	1.53	445.4
δ	0.51	1.61	281.6

**Figure 3:**
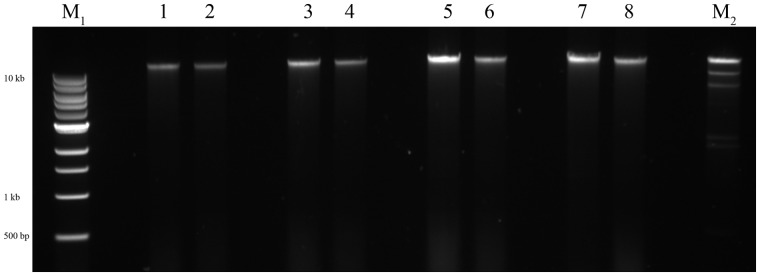
Extracts pre- and postethanol precipitation. The 1, 2, α crude and purified, respectively; 3, 4, β crude and purified; 5, 6, γ crude and purified; 7, 8, δ crude and purified. $M_{1}^{,}$ 1Kb ladder; $M_{2}^{,}$ Lambda *Hin*dIII digest (23 kb band, 47.7 ng DNA).

**Figure 4:**
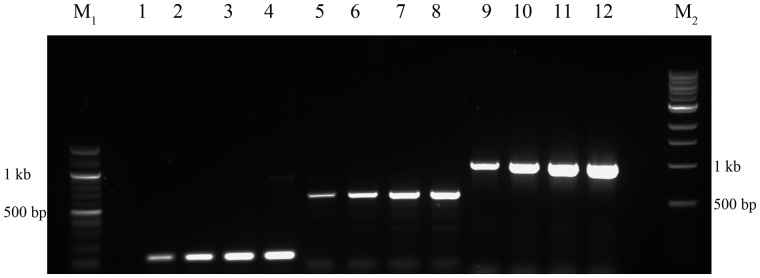
Replicate PCR using α, β, γ, and δ from purified extract as template, respectively. Lanes 1–4, PCR for 16S rRNA product; 5–8, PCR for 620 bp product; 9–12, PCR for 1024 bp product. $M_{1}^{,}$ 100 bp ladder, $M_{2}^{,}$ 1 kb ladder.

There was also good recovery of genomic DNA after purification, quantified by spectrophotometry (Table 2) with the desired improvement in A_260_/A_230_ and A_260_/A_280_ ratios in most cases (desired A_260_/A_280_ of 1.8 [26]). Gel electrophoresis of all samples, with and without purification steps (Fig. 3), showed large genomic DNA fragments and no smear (Fig. 3, Supplementary Fig. S3). Purified samples were tested in PCR as above, with all extracts now serving as templates for successful amplification (Fig. 4). This included PCR from stored (frozen) extractions (Supplementary Fig. S3). Digests were also successful with DNA from all extracts (Supplementary Fig. S3).

**Table 2:** DNA concentration and quality following rapid extraction with purification

		Quality following purification				
Sample name	Input DNA (μg)	A_260_/A_230_	A_260_/A_280_	Concentration (ng/μl)	Total recovery (μg)	Proportion recovery (%)
α	19.76	0.75	1.48	130.0	13.00	66
β	21.26	0.72	1.58	157.0	15.70	74
γ	44.54	0.79	1.68	176.0	17.60	40
δ	28.16	1.03	1.88	188.8	18.80	67

## Discussion

Numerous methods exist for cyanobacterial genomic DNA extraction which achieve high-quality samples suitable for sequencing. The standard use of high-quality cyanobacterial extracts, indicated by ratios of A_260_/A_230_ of 2.0 and A_260_/A_280_ of 1.8 [26, 27], is not necessary for PCR-based cloning, screening transformants, or early investigations. Cyanobacterial colony PCR is often refractory, and material cannot be retained for future PCR reactions. Here, DNA was quickly prepared from new and longstanding *Synechocystis* cultures, avoiding delicate, harmful, or expensive reagents such as chloroform, lysozyme, or kit columns. The optimum density of *Synechocystis* cultures for rapid extraction was ∼8.36 × 10^8^ c.f.u./ml but this was not critical.

The simplest method provided material effective as template for PCR in the majority of cases (Fig. 2). A further purification step could achieve samples with A_260_/A_280_ close to 1.8 although, even when there was little improvement in spectrophotometric purity, PCR was more successful (in the case of extract “α” which had the lowest A_260_/A_280_ and a low A_260_/A_230_ indicating residual carbohydrate contamination; Table 2; Figs 2 and 4). DNA visualized by gel electrophoresis revealed integrity of genomic DNA, suggesting minimal degradation. Digests were successful on all extracts, including fresh or frozen preparations, and could be useful for library construction, for example. Therefore, this is an inexpensive and straightforward method to produce and archive genetic material, which requires minimal equipment and reagents, and can start with any extant culture of this model cyanobacterium. This should aid all early studies in *Synechocystis* biology and biotechnology.

## Supplementary Material

bpaa011_Supplementary_Data
