# Supplementary material for: A rapid and low-cost method for genomic DNA extraction from the cyanobacterium Synechocystis
Source: Biol Methods Protoc. 2020 Jun 13;5(1):bpaa011. doi: 10.1093/biomethods/bpaa011 (PMC7474859; doi:10.1093/biomethods/bpaa011)
Supplement: bpaa011_Supplementary_Data [file bpaa011_supplementary_data.docx]

**Supplementary Data**

**
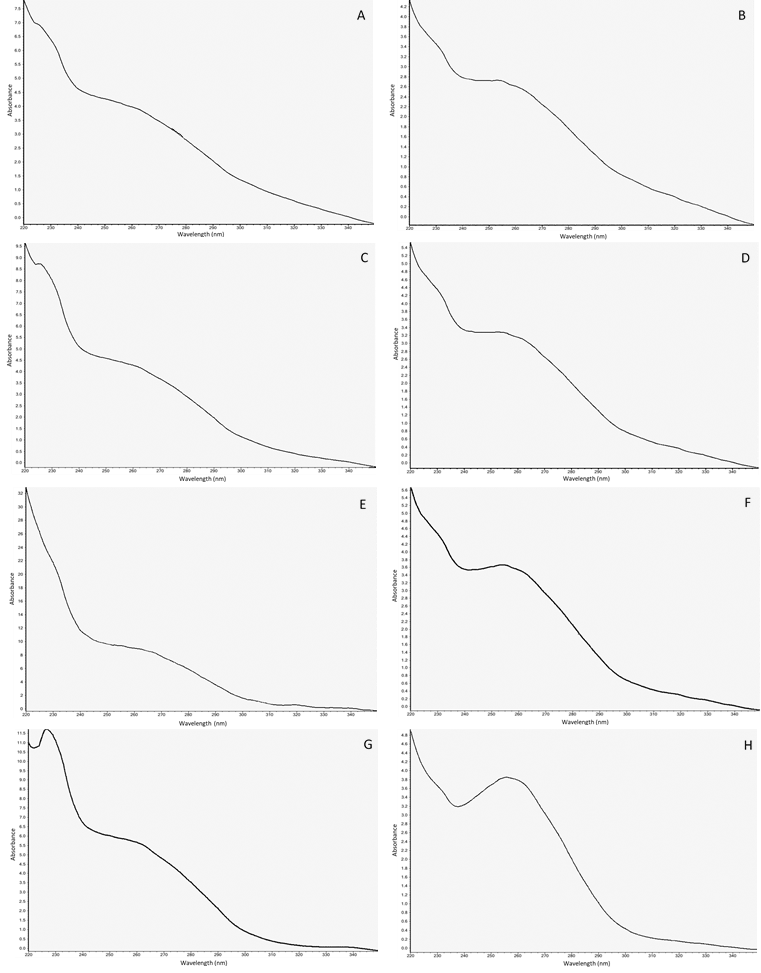
Suppl. Fig. 1.** Extracted *Synechocystis* DNA; A, α extract; B, α purified; C, β extract; D, β purified; E, γ extract; F, γ purified; G, δ extract; H, δ purified (ThermoFisher Nanodrop 2000c).

**
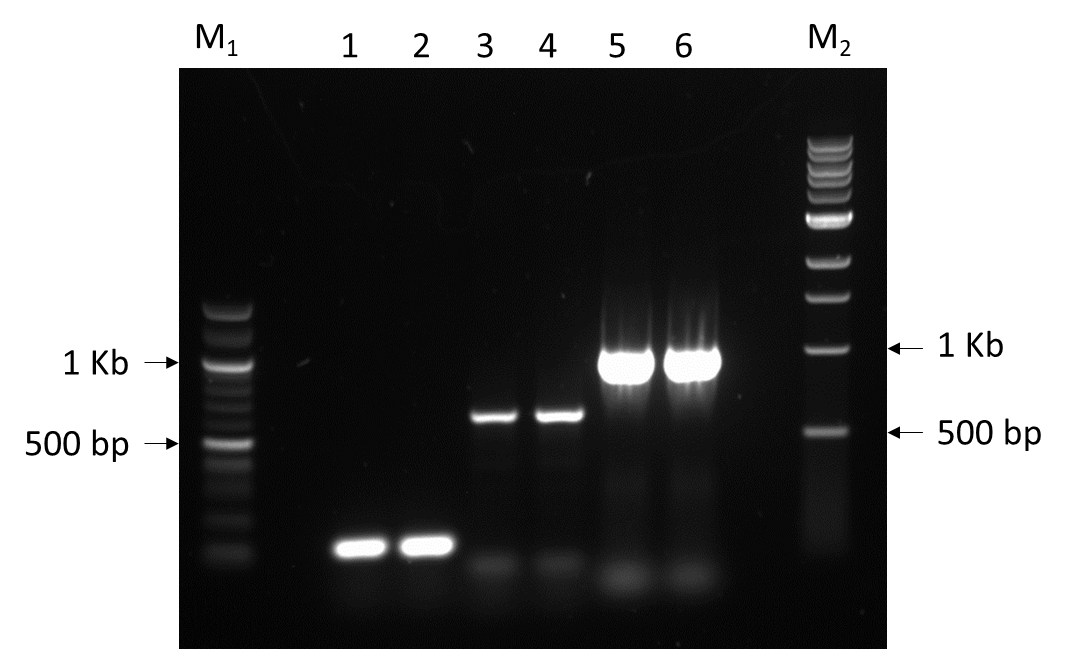
**

**Suppl. Fig. 2.** PCR using DNA from stock (old, not in exponential growth) versus new *Synechocystis* cultures: M1, 100bp ladder, 1, 2, old vs new 16S rRNA product; 3, 4, old vs new 620 bp product; 5, 6, old vs new 1024 bp product; M2, 1Kb ladder.

(A)

**
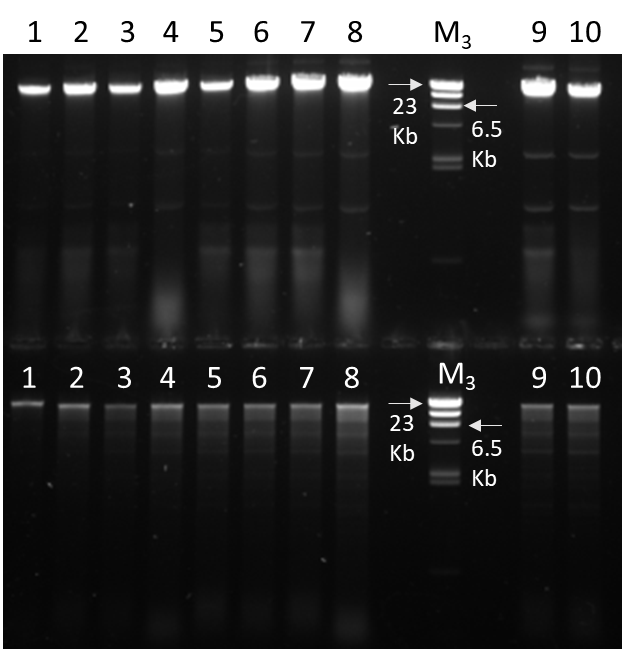
**

(B)

**
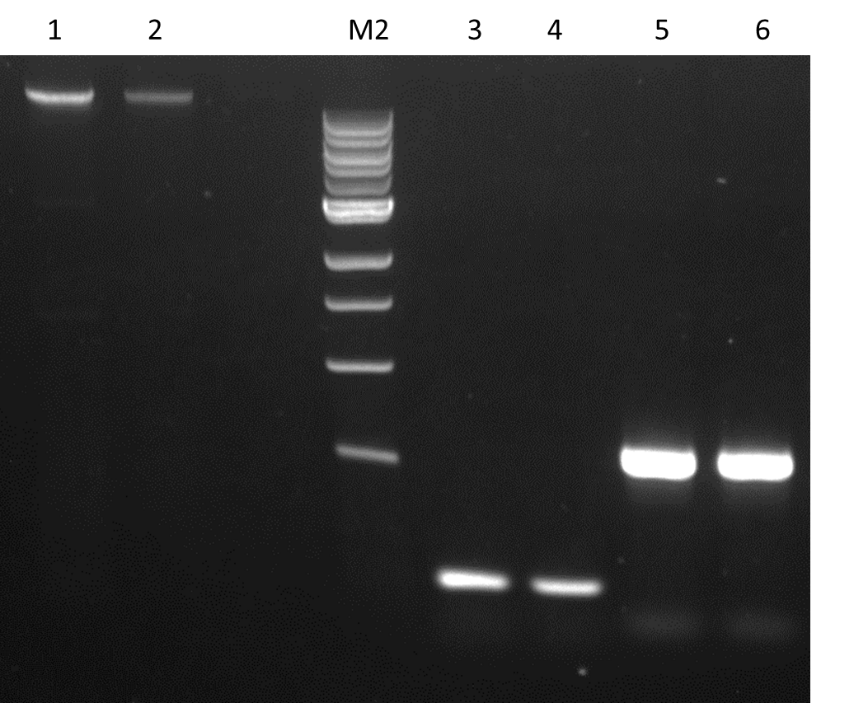
**

**Suppl. Fig. 3.** Use of extracts stored at -20degC. (A) Undigested controls (top wells) and *Nhe*I digests (lower wells) of new extracts (lanes 1, 3, 5, 7), and frozen, stored extracts (lanes 2, 4, 6, 8). Right panel, Stock culture (not in exponential growth) quick extract (lane 9), pure extract (lane 10): top, undigested; lower panel, *NheI* digests. (B) PCR: 1, fresh, 2, stored template; 3, 4, PCR with 16S rRNA primer pair for fresh and stored template; 5, 6, PCR for 620bp product with fresh and stored template. M2, 1 Kb ladder; M3, Lambda *Hin*dIII digest.
